# Analysis of reporting completeness in exercise cancer trials: a systematic review

**DOI:** 10.1186/s12874-019-0871-0

**Published:** 2019-12-02

**Authors:** Jose Francisco Meneses-Echavez, Indira Rodriguez-Prieto, Mark Elkins, Javier Martínez-Torres, Lien Nguyen, Julia Bidonde

**Affiliations:** 1 Division for Health Services, Norwegian Institute of Public Health, Sandakerveien 24C, Building D11, 4th floor, office, 434 Oslo, Norway; 2 Facultad de Cultura Física, Deporte y Recreación, Universidad Santo Tomás, Bogotá, Colombia; 3 Grupo de investigación Movimiento Corporal Humano, Universidad de la Sabana. Facultad de Enfermería y Rehabilitación, Chía, Colombia; 4 Sydney Medical School, Sydney, Australia; 5 Grupo “GRINMADE”, Facultad de Medicina, Universidad de Antioquia, Medellin, Colombia; 6 School of Rehabilitation Science, University of Saskatchewan, Saskatoon, Canada

**Keywords:** Exercise, Cancer, Reporting, Systematic review

## Abstract

**Background:**

Exercise is an effective therapeutic intervention for cancer survivors. Concerns about the completeness of reporting of exercise interventions have been raised in the literature, but without any formal analysis. This study aimed to evaluate the completeness of reporting of exercise interventions for cancer survivors in a large sample of randomized clinical trials (RCTs).

**Methods:**

We developed a pre-defined protocol. We searched MEDLINE, EMBASE, and CENTRAL for exercise trials in oncology between 2010 and 2017. Pairs of independent researchers screened the records, extracted study characteristics, and assessed 16 items on the TIDieR checklist (i.e., the 12 items, with item 5 divided into two and item 8 divided into four). For each of these items, the percentage of interventions in the included studies that reported the item was calculated.

**Results:**

We included 131 RCTs reporting 138 interventions in the analysis. Breast cancer was the most common type of cancer (69, 50%), and aerobic exercise was the most studied exercise modality (43, 30%) followed by combined aerobic and resistance training (40, 28%). Completeness of reporting ranged from 42 to 96% among the TIDieR items; none of the items was fully reported. ‘Intervention length’ was the most reported item across interventions (133, 96%), followed by ‘rationale’ (131, 95%), whereas ‘provider’ (58, 42%) and ‘how well (planned)’ (63, 46%) were the two least reported items. Half of the TIDieR items were completely reported in 50 to 70% of the interventions, and only four items were reported in more than 80% of the interventions (Items 2 and 8a to c). The seven items deemed to be core for replication (Items 3 to 9) exhibited a mean reporting of 71%, ranging from 42 to 96%.

**Conclusion:**

Exercise training interventions for cancer survivors are incompletely reported across RCTs published between 2010 and 2017. The reporting of information about the provider, materials, and modifications require urgent improvements. Stronger reporting will enhance usability of trial reports by both healthcare providers and survivors, and will help to reduce research waste.

## Background

Exercise is recognized as one of the most effective non-pharmacological interventions for improving outcomes for cancer survivors [1]. A strong body of evidence suggests that cancer survivors who exercise gain benefits in quality of life, fatigue, mobility [2], depression, post-operative outcomes [3], and the tumor microenvironment [4, 5]. The first report of the American Cancer Society about exercise and cancer was published in 2003 [6]; and since then, the number of randomized clinical trials (RCTs) addressing the effects of exercise in cancer survivors has grown exponentially.

The rapid accumulation of RCTs of exercise in cancer survivors should improve clinical outcomes, but only if the exercise interventions are reported thoroughly. Incomplete reporting of the exercise interventions impedes clinicians', researchers' and patients’ use of the evidence [7–10]. Incomplete reporting of interventions can also impair the synthesis of evidence (i.e. systematic reviews) in several ways [11, 12]: trials may be erroneously included or excluded because of uncertainty about the intervention; and treatment differences may go unrecognized as a source of between-study variation in effect estimates. By impairing systematic reviews, clinical decision-making is also affected [13, 14].

Complete reporting of interventions encompasses more than just naming or labelling the intervention and listing its main components; researchers must report also on other key features of the interventions, such as duration, intensity/dose, setting, mode of delivery, and monitoring [8, 9, 15, 16]. Reporting the rationale/framework underlying the intervention may aid clinicians to adjust it to suit the comorbidities or other characteristics of individual patients.

The completeness of reporting is generally lower in non-pharmacological than in pharmacological trials [8]. In reviews of trials of exercise in cancer survivors, various research groups have expressed concern about the description of the exercise protocols [1, 17, 18]. We found similar examples in the literature. In one review, only 39% of the non-pharmacological trials provided complete data for the intervention details [9]. In a review of supervised exercise training in people with peripheral arterial disease, only around one-quarter of the trials described complete data for the mode of exercise, intensity of exercise, and tailoring/progression; and around one-tenth reported exercise intensity comprehensively [19]. Similar findings were found on exercise-based cardiac rehabilitation trials [20]. The study by Candy on complex interventions in education and psychotherapies [21], concluded there was “no overall evidence that reporting the specifics of multicomponent, non-pharmacological interventions is improving”. In addition, Candy mentioned, “details to replicate interventions remain lacking, impairing best implementation or meaningful further research” [21].

In order to assess the completeness of reporting, various checklists have been developed; for example the SPIRIT (Standard Protocol Items: Recommendations for Interventional Trials) statement for use at the protocol stage [22] and the CONSORT (Consolidated Standards of Reporting Trials) for pharmacological [23] and non-pharmacological treatments [24] at the manuscript stage. The TIDieR (template for intervention description and replication) tool was published as an extension to the above documents, to increase the detail reported about interventions [25]. The checklist contains 12 items: name, why, what, who provided, how, where, when and how much, tailoring, modifications, how well, adherence and fidelity [25]. Authors conducting intervention RCTs are encouraged to use the TIDieR checklist to enable replication and facilitate the potential impact of their research on both health and society [25].

To our knowledge, no formal analysis has been published in the cancer and exercise field. The present study aimed to evaluate the completeness of reporting of exercise training interventions in RCTs that test exercise interventions in cancer survivors, using items on the TIDieR checklist.

## Methods

This study is reported according to the PRISMA statement [26] and the guidelines for reporting meta-epidemiological methodology research [27]. We developed the protocol a priori and made it available via Open Science Framework (https://osf.io/6ejh9/?view_only=4320d9fbe4134ca88422d1eaf3d5b44a; DOI 10.17605/OSF.IO/6EJH9). We present amendments made to the protocol in Additional file 1.

### Search strategy and screening

**Fig. 1:**
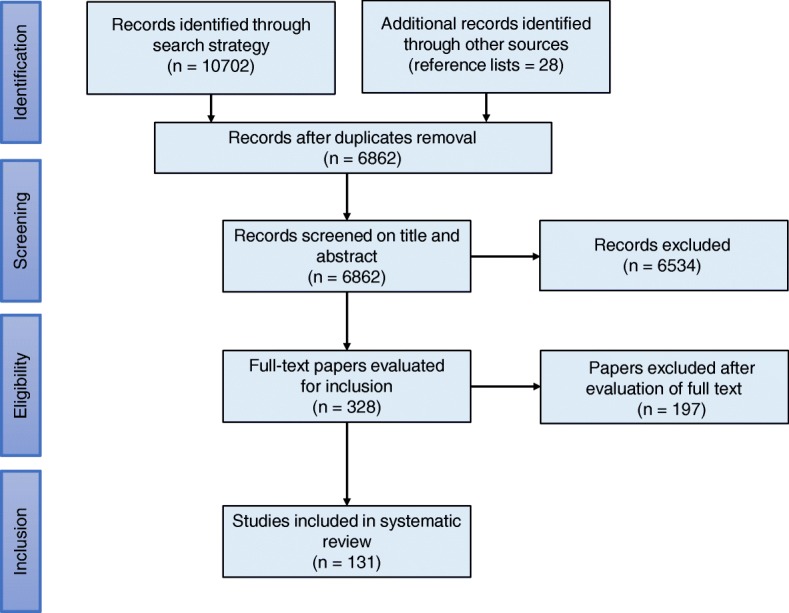
Flow diagram for the selection of the studies

An information specialist (LN) designed, tested and implemented a systematic search for RCTs published in the MEDLINE, EMBASE, and CENTRAL databases between 2010 and 2017. We selected 2010 because of the most recent update of the CONSORT statement [23], which was launched in 2010. One reviewer (JME) screened the reference lists of relevant systematic reviews in the field. Additional file 2 presents the search strategies for MEDLINE, EMBASE and CENTRAL. We used the management software Rayyan [28] for independent screening of title and abstract. Pairs of researchers discussed disagreements, with resolution by an independent third researcher when necessary. Figure 1 presents the flow of information through the different phases of a systematic review.

### Selection criteria

We included RCTs meeting the following criteria:

### Population

Adult (older than 18 years old) survivors of any type of cancer. A survivor was defined according to the Centers for Disease Control and Prevention (CDC), as anyone who has been diagnosed with cancer, from the time of diagnosis through the rest of life [29].

### Intervention

RCTs evaluating the effects of exercise training interventions for cancer survivors. Exercise training was defined as any body movement that increases energy expenditure and that is planned, structured, repetitive, and purposive in the sense that it aims to improve or maintain one or more components of physical fitness (i.e., cardiorespiratory endurance, muscular endurance, muscular strength, body composition, and flexibility) [30, 31].

We accepted for inclusion any exercise training interventions involving different training modes, such as aerobic, resistance and flexibility training, as well as yoga, Qi-gong and Tai-Chi [32, 33]. Further, the exercise training interventions could be conducted in different settings (such as clinical or community) or mediums (such as water or land). Because of the review’s focus on the reporting of exercise training interventions, we excluded RCTs that evaluated recreational physical activity interventions, as well as trials reporting on manual therapy (e.g., joint mobilization techniques and therapeutic massage), cognitive-behavioral interventions, and mixed interventions that combined exercise with other therapeutic approaches, such as psychotherapy or diet or dietary advice/counseling. Finally, we excluded trials that compared exercise training with pharmacological and surgical treatments.

### Comparison

We included studies with non-exercise intervention comparisons (such as conventional care) or other exercise interventions (e.g., aerobic versus resistance training). Where trials compared two exercise interventions, both interventions were included in the analysis.

### Study design and type of publication

We included RCTs. If there was any dispute about the eligibility of a trial’s design, we referred to the National Cancer Institute’s definition [34]. Only full-text publications were included in the review. As we included studies and not research papers, when multiple publications from a single RCT were found, authors decided to use the primary publication for this analysis. We made this decision to avoid double counting of studies and for practical reasons.

### Language

We considered for inclusion studies published in languages the team could translate i.e. English, Spanish, Italian, Portuguese and Scandinavian languages.

### Data extraction and management

#### Characteristics of the included studies

We extracted the following information: publication year, country, trial registry, study name, primary publication/companion, sample size (total analyzed), type of cancer, treatment stage, control group(s), exercise mode, length (weeks, with the minimum value reported in case of range), frequency (sessions/week), and setting. Walking interventions were classified as aerobic exercise.

#### TIDieR checklist and calculation of completeness of reporting

A pair of researchers (JME, JB, IR, JMT) worked independently to apply the TIDieR checklist to the included RCTs. As recommended by the TIDieR committee, the checklist is completed following the TIDieR guide [25], which contains an explanation and elaboration for each item. All the items were rated Yes/No. Only items that were clearly met were rated Yes; any that were partially met were rated No. Pairs of researchers discussed disagreements, with those outstanding resolved by an independent third researcher. Item 5 contributed two components and Item 8 contributed four components. See Table 1.

**Table 1:** Final version of the TIDieR checklist used in this study (16 items)

1. Brief name	
Provide the name or a phrase that describes the intervention	
2. Why	
Describe any rationale, theory, or goal of the elements essential to the intervention	
3. What (Materials)	
Describe any physical or informational materials used in the intervention	
4. What (procedures)	
Describe each of the procedures, activities, and/or processes used in the intervention	
5. a. Who provided (disciplinary background)	
Describe the disciplinary background of the provider	
b. Who provided (expertise, experience, or specific training)	
Describe the expertise, experience, or specific training of the provider	
6. How	
Describe the modes of delivery	
7. Where	
Describe the type(s) of location(s) where the intervention occurred	
8. a. When and how much (frequency)	
Describe the number of times the intervention was delivered (e.g., number of sessions)	
b. When and how much (length)	
Describe the number of weeks/months the intervention lasted	
c. When and how much (duration)	
Describe the duration of each session (e.g., minutes /session)	
d. When and how much (intensity)	
Describe the intensity at which the exercise was practiced	
9. Tailoring	
If the intervention was planned to be personalized, titrated or adapted, then describe what, why, when, and how	
10. Modifications	
If the intervention was modified during the course of the study, describe the changes	
11. How well (planned)	
If intervention adherence or fidelity was assessed, describe how and by whom, and if any strategies were used to maintain or improve fidelity, describe them.	
12. How well (actual)	
If intervention adherence or fidelity was assessed, describe the extent to which the intervention was delivered as planned.	

#### Overall and subgroup analyses

For each of these items, the percentage of interventions in the included studies that reported the item was calculated. We presented separate data for the subgroups of breast cancer and non-breast cancer trials, and exercise modality.

## Results

### Results of the search

The systematic searches yielded 10,702 records, and 28 additional records were found by hand searching systematic reviews in this field. After removal of duplicates, we exported 6862 records to Rayyan for screening of title and abstract, after which we read 328 records as full-text manuscripts. One reviewer (JME) retrieved all full-text publications. We included 131 RCTs in our analysis.

### Characteristics of the included studies

The 131 RCTs contributed information about 138 interventions to the analysis. The characteristics of the individual included studies/interventions such as country, year of publication, sample size, type of cancer reported, treatment stage, and other are presented in Additional file 3. Summary data are presented below.

Twenty-one trials (16%) provided study name or acronym, and forty-four (34%) reported their trial registry record/trial protocol. Hereafter we refer to interventions (rather than trials) as they represent our unit of analysis.

Overall, we report data from 38 countries. USA was the most common country across the analyzed interventions (38, 27%), followed by Australia (17, 12%), Canada (12, 9%), Germany (11, 8%), Korea (6, 4%), and Spain (5, 4%). Around half of the interventions were performed in groups of 10 to 50 participants (65, 47%), and one-third included 51 to 100 participants (47, 34%). Breast cancer was the most common type of cancer (69, 50%), followed by prostate cancer (20, 14%), mixed (more than one type) (14, 10%), and colorectal cancer (7, 5%). Most exercise interventions were administered to people receiving active cancer treatment (71, 51%), followed by post-treatment administration (62, 45%) and pre-operative administration (6, 4%).

#### Interventions: exercise modalities

Aerobic exercise was the most studied exercise modality (43, 30%) followed by combined aerobic/resistance training (40, 28%). Resistance training alone and yoga accounted for around 13% of the interventions each. Other modalities comprised Qigong, aquatic exercise, football, high-intensity training, and Tai-Chi. One-third of the exercise interventions were implemented in clinics or hospitals. On average, exercise interventions lasted 14.3 weeks (range 1 to 104 weeks), and involved 2.8 (range 1 to 14) sessions per week.

### Completeness of reporting of the exercise training interventions

#### Total sample

**Fig. 2:**
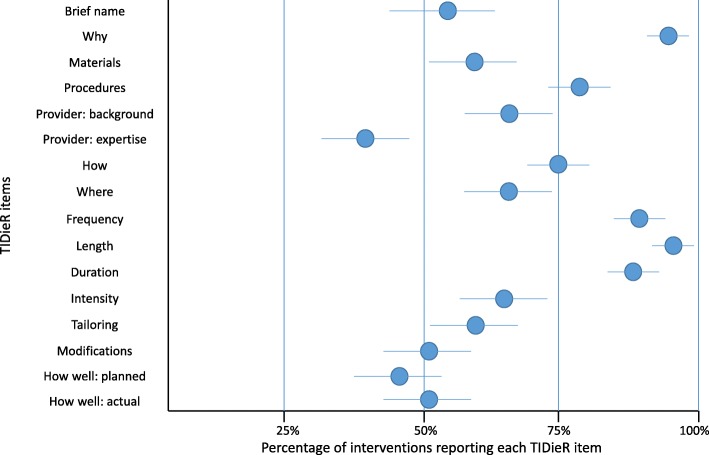
Completeness of reporting of the exercise interventions: total sample (*n* 131 RCTs; 138 exercise interventions)

Completeness of reporting ranged from 42 to 96% among the TIDieR items (see Fig. 2). *Intervention length* was the most reported item across interventions (133, 96%), followed by *study rationale* (131, 95%), whereas *provider* (58, 42%) and *how well (planned)* item (63, 46%) were the two least reported items. Half of the TIDieR items were reported in 50 to 70% of the interventions, and only four items were reported in more than 80% of the interventions (items 2 and 8a-c). In addition, the seven items [3–9] deemed to be core for replication [25] exhibited a mean reporting of 71%, ranging from 42 to 96%.

#### Subgroup analysis 1: breast cancer

Breast cancer exercise-training interventions counted for half of the total sample in this study. However, completeness of reporting among the interventions exhibited similar results to the total sample. The largest difference was a 17% less complete reporting of item 7 (*where*) than in the non-breast cancer subgroup. See Table 2.

**Table 2:** Completeness of reporting of the exercise interventions: total sample and type of cancer subgroups

TIDieR item	Total sample n (%)	Breast cancer n (%)	Non-breast cancer n (%)
Item 1. Brief name	76 (55%)	36 (52%)	40 (58%)
Item 2. Why	131 (95%)	66 (96%)	65 (94%)
Item 3. What (materials)	81 (59%)	40 (58%)	41 (59%)
Item 4. What (procedures)	108 (78%)	53 (77%)	55 (80%)
Item 5a. Who provided (disciplinary background)	93 (67%)	44 (64%)	49 (71%)
Item 5b. Who provided (expertise, experience, or specific training)	58 (42%)	30 (44%)	28 (41%)
Item 6. How	104 (75%)	54 (78%)	50 (72%)
Item 7. Where	92 (67%)	40 (58%)	52 (75%)
Item 8a. Frequency	123 (89%)	60 (87%)	63 (91%)
Item 8b. Length	133 (96%)	66 (96%)	67 (97%)
Item 8c. Duration	120 (87%)	63 (91%)	57 (83%)
Item 8d. Intensity	90 (65%)	39 (56%)	51 (74%)
Item 9. Tailoring	82 (59%)	37 (54%)	45 (65%)
Item 10. Modifications	70 (51%)	32 (46%)	38 (55%)
Item 11. How well (planned)	63 (46%)	30 (43%)	33 (48%)
Item 12. How well (actual)	70 (51%)	35 (51%)	35 (51%)

#### Subgroup analysis 2: non-breast cancer

The other half of the interventions, those that involved non-breast cancer patients, comprised predominantly prostate cancer (20, 29%), followed by mixed type and lung cancers (each 14, 20%), and colorectal cancer (7, 10%). In general, this subgroup was reported in a more complete manner than the breast cancer subgroup and the total sample. Only a few items showed lower scores of complete reporting relative to the other groups (Item 2, item 5b, item 6, and item 8c). See Table 2.

#### Subgroup analysis 3: exercise modality

Overall, aerobic exercise plus resistance training interventions had the lowest level of reporting relative to the other two subgroups (i.e., aerobic exercise and resistance training). Item 2 (why), item 8a (frequency), and item 8b (length) were completely reported in more than 90% of the interventions in all three subgroups. The last two items (items 8 a and b) reached 100% reporting in resistance training interventions. Item 5b (who provided, expertise) was the least reported item across the subgroups. See Table 3.

**Table 3:** Completeness of reporting of by exercise modality: aerobic exercise; aerobic exercise plus resistance training; resistance training

TIDieR item	Aerobic exercise (*n* = 43)	Aerobic exercise plus resistance training (*n* = 40)	Resistance training (*n* = 18)
	**n (%)**	**n (%)**	**n (%)**
Item 1. Brief name	31 (72.1%)	18 (45%)	10 (55.6%)
Item 2. Why	40 (93%)	37 (92.5%)	18 (100%)
Item 3. What (materials)	29 (67.4%)	17 (42.5%)	11 (61.1%)
Item 4. What (procedures)	34 (79.1%)	29 (72.5%)	16 (88.9%)
Item 5a. Who provided (disciplinary background)	32 (74.4%)	20 (50%)	10 (55.6%)
Item 5b. Who provided (expertise. experience. or specific training)	17 (39.5%)	12 (30%)	5 (27.8%)
Item 6. How	31 (72.1%)	26 (65%)	13 (72.2%)
Item 7. Where	35 (81.4%)	23 (57.5%)	10 (55.6%)
Item 8a. Frequency	40 (93%)	36 (90%)	18 (100%)
Item 8b. Length	40 (93%)	38 (95%)	18 (100%)
Item 8c. Duration	37 (86%)	32 (80%)	16 (88.9%)
Item 8d. Intensity	35 (81.4%)	34 (85%)	14 (77.8%)
Item 9. Tailoring	29 (67.4%)	23 (57.5%)	14 (77.8%)
Item 10. Modifications	15 (34.9%)	21 (53.8%)	15 (83.3%)
Item 11. How well (planned)	20 (46.5%)	18 (46.2%)	10 (55.6%)
Item 12. How well (actual)	24 (55.8%)	15 (39.5%)	12 (66.7%)

## Discussion

### Main findings

This study evaluated the completeness of reporting of exercise training interventions in a sample of RCTs in cancer survivors. Findings revealed none of the TIDieR items was fully reported across all the interventions in the RCTs. Intervention length and study rationale were the two most reported items. Conversely, relevant information for researchers, healthcare providers, and patients (such as the expertise, experience, or specific training of the provider) obtained the lowest score of reporting. We observed no major differences in the subgroups of breast cancer and non-breast cancer trials, but aerobic exercise and resistance training interventions had the lowest level of reporting compared to the groups of aerobic exercise and resistance training.

### Comparison with previous studies

To our knowledge, this is the first study addressing the completeness of reporting in exercise trials involving cancer patients, by using the TIDieR checklist. Other studies have applied TIDieR to exercise trials in people with peripheral arterial disease (58 trials, reporting on 76 interventions) [19], trials on exercise-based cardiac rehabilitation (57 trials, reporting on 74 interventions) [20], and in trials of upper limb therapies for children with unilateral cerebral palsy (60 trials, reporting on 68 interventions) [35]. In the field of exercise and cancer, Neil-Sztramko and collaborators have recently conducted relevant work about the reporting of the components and principles of resistance training prescription in breast cancer trials that measured physical fitness or body composition outcomes [36]. That study found that no trials reported all components of the exercise prescription in the methods, or adherence to the prescribed intervention in the results. Similar findings were found in prostate cancer trials [37].

### Strengths and weaknesses

The systematic search run for this study as well as the independent and duplicate conduct of the study selection and data extraction processes constitute methodological strengths. The large number of RCTs evaluated represent the largest study in using the TIDieR checklist to date. Moreover, the research team comprised a journal editor, healthcare providers, and experts in evidence synthesis in the area of exercise in cancer and other chronic conditions. We believe the decision of splitting the number of TIDieR items in our analysis provides a more specific insight to readers interested in this field. The developers of the TIDieR tool supported this approach.

In the event of a single study being reported in multiple publications we chose the primary publication for our analysis. This decision may be a limitation as more complete reporting could have been included in the companion articles. We did this for pragmatic reasons and because we think it is reasonable to expect that the intervention is thoroughly described in the primary report of the study.

### Implications for practice and further research

Findings from this study encourage researchers to adhere to international reporting guidance when formulating and publishing their research in order to facilitate translations of their findings into practice. Our results indicate there is still work to be done in this regard. A better reporting of exercise interventions facilitates evidence uptake by clinicians, patients and decision-makers. Besides, a complete reporting of exercise interventions and further research on the reporting of research is one of the key strategies in the battle against research waste [8, 38, 39]. As recently stated by Glasziou and colleagues [39], “unless research is adequately reported, the time and resources invested in the conduct of research is wasted.”

Another purported benefit of reporting checklists such as TIDieR is that they facilitate replication. Further research could examine original and replication trials to determine the completeness of reporting and the faithfulness of the replication.

Future research could examine the reporting of some additional items, some of which have more recently been listed in the Consensus on Exercise Reporting Template (CERT) tool [40]. These factors include whether the exercise is supervised and whether motivational strategies are used. We did not know about the CERT tool at the time our study protocol was formulated, and decided that it was not worth changing the study protocol to incorporate the CERT a posteriori, because of the substantial overlap between it and TIDieR. Another reporting guideline of interest to health professionals in this area is CReDECI 2 (Criteria for Reporting the Development and Evaluation of Complex Interventions in healthcare) [41], which focus on complex interventions. Although not all cancer exercise interventions are complex [42], the CReDECI guidelines may provide an alternative checklist for reporting of experimental studies.

Journals should encourage trial authors to adhere to reporting guidance when processing submissions. Thus, journals should endorse checklists for reporting interventions as they do for CONSORT or any other related statements [25]. Hopewell et al. [43] found in a time series design that an active implementation of the CONSORT for abstracts guidelines by journals improved the number of checklist items reported in abstracts of randomized trials. Journals might ask researchers to use TIDieR and perhaps CERT in conjunction when completing item 5 of the CONSORT checklist, and there refer the reader to a detailed assessment of the intervention-reporting checklist.

## Conclusion

Exercise training interventions for cancer survivors are reported moderately well among RCTs published between 2010 and 2017. The reporting of information about the provider, materials, and modifications requires urgent improvement. More complete reporting of exercise training interventions for cancer patients will enhance trial usability for both healthcare providers and patients, and will contribute to a large extent in the battle to reduce research waste. Researchers might use the TIDieR checklist when reporting their exercise interventions in further trials.

## Supplementary information

**Additional file 1.** Amendments to the protocol. This file contains the amendments made to the study protocol

**Additional file 2.** Search strategy. This file presents the search strategies used to identify the individual studies

**Additional file 3 **Characteristics of the included studies (*n* 131 RCTs; 138 exercise interventions). This file presents a detailed overview of the characteristics of the included studies

## Data Availability

The datasets used and/or analyzed during the current study are available from the corresponding author on reasonable request.

## Abbreviations

CERT: Consensus on Exercise Reporting Template
CONSORT: Consolidated Standards of Reporting Trials
CReDECI 2: Criteria for Reporting the Development and Evaluation of Complex Interventions in healthcare
PRISMA: Preferred Reporting Items for Systematic Reviews and Meta-Analyses
RCT: Randomized Clinical Trial
SPIRIT: Standard Protocol Items: Recommendations for Interventional Trials
TIDieR: Template for Intervention Description and Replication
