# Supplementary material for: Analysis of reporting completeness in exercise cancer trials: a systematic review
Source: BMC Med Res Methodol. 2019 Dec 2;19:220. doi: 10.1186/s12874-019-0871-0 (PMC6889190; doi:10.1186/s12874-019-0871-0)
Supplement: Supplementary file 1 — Additional file 1. Amendments to the protocol. This file contains the amendments made to the study protocol [file 12874_2019_871_MOESM1_ESM.docx]

**Additional files**

**Additional file 1.** Amendments to the protocol

| **Study section (as stated in the protocol)** | **Amendment** |
| --- | --- |
| Completeness of reporting in the trials will be calculated as the number of the 12 criteria met, and presented using descriptive statistics | We calculated completeness of reporting in the RCTs for each TIDieR item and expressed them as percentages. |
| We will present separate data for the subgroups of breast cancer and non-breast cancer trials. | We presented the following subgroups: breast cancer and non-breast cancer trials as well as exercise modality.  The last subgroup was added after suggestion by the reviewers |
