# Supplementary material for: Analysis of reporting completeness in exercise cancer trials: a systematic review
Source: BMC Med Res Methodol. 2019 Dec 2;19:220. doi: 10.1186/s12874-019-0871-0 (PMC6889190; doi:10.1186/s12874-019-0871-0)
Supplement: Supplementary file 2 — Additional file 2. Search strategy. This file presents the search strategies used to identify the individual studies [file 12874_2019_871_MOESM2_ESM.docx]

**Additional file 2.** Search strategy

**Database(s): Ovid MEDLINE(R) Epub Ahead of Print, In-Process & Other Non-Indexed Citations, Ovid MEDLINE(R) Daily and Ovid MEDLINE(R) 1946 to Present**

**Date: 27.10.2017**

**Hits: 4273**

# Searches Results

1 exp Neoplasms/ 3185126

2 (cancer* or tumor* or tumour* or neoplasm* or leukaemi* or leukemi* or mesothelioma or myeloma* or lymphoma* or melanoma* or sarcoma* or myelomatos* or malignan*).tw. 3079834

3 1 or 2 4029228

4 exp Exercise/ 169977

5 exp Exercise Therapy/ 43909

6 exercise movement techniques/ 619

7 breathing exercises/ 3211

8 dance therapy/ 291

9 exp Sports/ 170511

10 Dancing/ 2641

11 Hydrotherapy/ 2475

12 Physical Fitness/ 27006

13 "Physical Education and Training"/ 14236

14 (physical* activit* or exercise* or training).tw. 634644

15 (yoga or pilates or swimming or running or jogging or walking or physical conditioning or motion therap* or movement therap* or stretching or weight* lifting or weight* bearing or muscle strengthening or power lifting or cycling or sport* or movement technique* or hydrotherap* or dancing or dance therap* or gymnastics or physical fitness or physical exertion* or calisthenic* or plyometric* or cardiopulmonary conditioning or sport* or ((resistance or strength or flexibility or endurance) adj6 progam*)).tw. 297556

16 or/4-15 950632

17 3 and 16 48847

18 randomized controlled trial.pt. 497817

19 controlled clinical trial.pt. 99283

20 randomized.ab. 434337

21 clinical trials as topic.sh. 195722

22 randomly.ab. 299368

23 trial.ti. 196126

24 or/18-23 1171590

25 exp animals/ not humans.sh. 4682440

26 24 not 25 1084894

27 17 and 26 5611

28 limit 27 to yr="2003-current" 4834

29 remove duplicates from 28 4273

**Database(s): Ovid EMBASE 1974 to 2017 October 26**

**Date: 27.10.2017**

**Hits: 1531**

# Searches Results

1 exp *neoplasm/ 3033716

2 (cancer* or tumor* or tumour* or neoplasm* or leukaemi* or leukemi* or mesothelioma or myeloma* or lymphoma* or melanoma* or sarcoma* or myelomatos* or malignan*).tw. 3825990

3 1 or 2 4493467

4 exp *exercise/ 127082

5 exp *physical activity/ 104518

6 *training/ 18673

7 *endurance/ 6378

8 exp *sport/ 64314

9 *hydrotherapy/ 1888

10 *dancing/ 1752

11 (yoga or pilates or swimming or running or jogging or walking or physical conditioning or motion therap* or movement therap* or stretching or weight* lifting or weight* bearing or muscle strengthening or power lifting or cycling or sport* or movement technique* or hydrotherap* or dancing or dance therap* or gymnastics or physical fitness or physical exertion* or calisthenic* or plyometric* or cardiopulmonary conditioning or sport* or ((resistance or strength or flexibility or endurance) adj6 progam*)).tw. 344237

12 or/4-11 540695

13 3 and 12 23429

14 crossover procedure/ 53868

15 double blind procedure/ 144512

16 randomized controlled trial/ 478919

17 single blind procedure/ 29996

18 (random* or factorial* or crossover* or cross over* or doubl* blind* or (singl* adj1 blind*) or assign* or allocat* or volunteer*).ti,ab. 1791790

19 14 or 15 or 16 or 17 or 18 1891467

20 13 and 19 3751

21 exp animals/ or exp invertebrate/ or animal experiment/ or animal model/ or animal tissue/ or animal cell/ or nonhuman/ 25463530

22 human/ or normal human/ or human cell/ 19171957

23 21 not (21 and 22) 6338890

24 (news or editorial or comment).pt. 551035

25 20 not (23 or 24) 3465

26 limit 25 to embase 1869

27 limit 26 to yr="2003-current" 1660

28 remove duplicates from 27 1531

**Database(s): CENTRAL (Cochrane Library)**

**Date: 27.10.2017**

**Hits: 4898**

ID Search Hits

#1 [mh Neoplasms] 62532

#2 (cancer* or tumor* or tumour* or neoplasm* or leukaemi* or leukemi* or mesothelioma or myeloma* or lymphoma* or melanoma* or sarcoma* or myelomatos* or malignan*):ti,ab,kw 145899

#3 #1 or #2 148503

#4 [mh Exercise] 19578

#5 [mh "Exercise Therapy"] 10844

#6 [mh ^"exercise movement techniques"] 186

#7 [mh ^"breathing exercises"] 698

#8 [mh ^"dance therapy"] 64

#9 [mh Sports] 13293

#10 [mh ^Dancing] 138

#11 [mh ^Hydrotherapy] 197

#12 [mh ^"Physical Fitness"] 2697

#13 [mh "Physical Education and Training"] 1526

#14 (physical* next activit* or exercise* or training):ti,ab,kw 95029

#15 (yoga or pilates or swimming or running or jogging or walking or physical next conditioning or motion next therap* or movement next therap* or stretching or weight* next lifting or weight* next bearing or muscle next strengthening or power next lifting or cycling or sport* or movement next technique* or hydrotherap* or dancing or dance next therap* or gymnastics or physical next fitness or physical next exertion* or calisthenic* or plyometric* or cardiopulmonary next conditioning or sport* or ((resistance or strength or flexibility or endurance) near/6 progam*)):ti,ab,kw 34717

#16 (44-#15)s 107689

#17 #3 and #16 Publication Year from 2003 to 2017 in Trials 4898
