# Supplementary material for: Analysis of reporting completeness in exercise cancer trials: a systematic review
Source: BMC Med Res Methodol. 2019 Dec 2;19:220. doi: 10.1186/s12874-019-0871-0 (PMC6889190; doi:10.1186/s12874-019-0871-0)
Supplement: Supplementary file 3 — Additional file 3 Characteristics of the included studies (n 131 RCTs; 138 exercise interventions). This file presents a detailed overview of the characteristics of the included studies [file 12874_2019_871_MOESM3_ESM.docx]

**Additional file 3.** Characteristics of the included studies (n = 131 RCTs; 138 exercise interventions)

| **Variables** | **N** | **%** |
| --- | --- | --- |
| **Publication year (n = 138)** | | |
| 2011 | 8 | 5.8% |
| 2012 | 14 | 10.1% |
| 2013 | 21 | 15.2% |
| 2014 | 25 | 18.1% |
| 2015 | 19 | 13.8% |
| 2016 | 30 | 21.7% |
| 2017 | 18 | 13% |
| 2018 | 3 | 2.2% |
| **Country (n = 131)** | | |
| USA | 38 | 29% |
| Australia | 17 | 13% |
| Canada | 12 | 9% |
| Germany | 11 | 8% |
| South Korea | 8 | 6.1% |
| Spain | 5 | 3.8% |
| China | 4 | 3% |
| Iran | 4 | 3% |
| Netherlands | 4 | 3% |
| Taiwan | 4 | 3% |
| United Kingdom | 4 | 3% |
| Denmark | 3 | 2.3% |
| Turkey | 3 | 2.3% |
| Italy | 2 | 1.5% |
| Sweden | 2 | 1.5% |
| Belgium | 1 | 0.7% |
| Belgrade | 1 | 0.7% |
| Brazil | 1 | 0.7% |
| Finland | 1 | 0.7% |
| France | 1 | 0.7% |
| India | 1 | 0.7% |
| Japan | 1 | 0.7% |
| Kosovo | 1 | 0.7% |
| Malaysia | 1 | 0.7% |
| Nigeria | 1 | 0.7% |
| Norway | 1 | 0.7% |
| Poland | 1 | 0.7% |
| Switzerland | 1 | 0.7% |
| Thailand | 1 | 0.7% |
| **Sample size (n = 138)** | | |
| 10-50 | 65 | 47.1% |
| 51-100 | 47 | 34.1% |
| 101-200 | 20 | 14.5% |
| 201-300 | 4 | 2.9% |
| 410 | 1 | 0.7% |
| 500 | 1 | 0.7% |
| **Type of cancer (n = 138)** | | |
| Breast | 69 | 50% |
| Prostate | 20 | 14.5% |
| General | 14 | 10% |
| Lung | 14 | 10% |
| Colorectal | 7 | 5.1% |
| Leukemia | 4 | 3% |
| Lymphoma | 2 | 1.4% |
| Bladder | 1 | 0.7% |
| Brain | 1 | 0.7% |
| Breast and Prostate | 1 | 0.7% |
| Gynecologic | 1 | 0.7% |
| Head and Neck | 1 | 0.7% |
| Lung and Colorectal | 1 | 0.7% |
| Pancreas | 1 | 0.7% |
| Testicular | 1 | 0.7% |
| **Treatment stage (n = 138)** | | |
| During treatment | 71 | 51.4% |
| Post-treatment | 62 | 45% |
| Preoperative | 5 | 3.6% |
| **Exercise modality (n = 138)** | | |
| Aerobic | 43 | 31.1% |
| Aerobic + Resistance | 40 | 28.9% |
| Resistance | 18 | 13% |
| Yoga | 18 | 13% |
| Qigong | 5 | 3.6% |
| Aquatic exercise | 3 | 2.2% |
| High-intensity training | 3 | 2.2% |
| Tai-Chi | 3 | 2.2% |
| Pilates | 2 | 1.4% |
| Stretching | 2 | 1.4% |
| Aerobic + Yoga | 1 | 0.7% |
| Dancing | 1 | 0.7% |
| Football | 1 | 0.7% |
| Tai-Chi + QiGong + Yoga | 1 | 0.7% |
| **Setting (n = 138)** | | |
| Clinic/hospital | 44 | 31.8% |
| Home | 30 | 21.7% |
| Mixed (any combination) | 27 | 19.5% |
| Not reported | 25 | 18.1% |
| Faculty lab | 15 | 10.8% |
